# Supplementary material for: Content-rich biological network constructed by mining PubMed abstracts
Source: BMC Bioinformatics. 2004 Oct 8;5:147. doi: 10.1186/1471-2105-5-147 (PMC528731; doi:10.1186/1471-2105-5-147)
Supplement: Additional File 2 — The original results of the above study (non-essential files are deleted to keep the file size under the limit set by BMC bioinformatics). [file 1471-2105-5-147-S2.bz2 › chilibotAdditionalFile2/dip20/563ID9374547E10924/html/MST1_MST1R.html]

 


 **MST1** and **MST1R** 
  
Found 69 abstracts in PubMed, retrieved 20.  
 

 What does Google say? 
 PDF only 
| .edu only 

---

**Interactive relationship** (e.g. stimulation, inhibition, etc)

**Stimulatory relationship**- The cytokine Macrophage Stimulating 1  [ **MST1** ]   **MST1**  MSP  [ **MST1** ]  Hepatocyte Growth Factor Like  [ **MST1** ]  is a ligand of the Met related  **MST1**  Receptor  **MST1R**  RON  [ **MST1R** ] .  Ref: 12764615 Dev Genes Evol< MedlineTA>Dev Genes Evol,
- RON  [ **MST1R** ]  is a receptor tyrosine kinase activated by macrophage stimulating protein  [ **MST1** ] .  Ref: 11818458 J Leukoc Biol, 2002
- The ligand of RON  [ **MST1R** ]  was identified as macrophage stimulating protein  [ **MST1** ]  MSP  [ **MST1** ] , a member of the plasminogen related growth factor family.  Ref: 12807733 Carcinogenesis< MedlineTA>Carcinogenesis, 2002
- Macrophage stimulating protein  [ **MST1** ]  MSP  [ **MST1** ]  is a pleiotropic growth factor that signals via the Ron  [ **MST1R** ]  receptor tyrosine kinase.  Ref: 12538524 Development, 2003
- In RON  [ **MST1R** ]  expressing macrophages treated with macrophage stimulating protein  [ **MST1** ] , LPS induced prostaglandin E 2 PGE 2 production was significantly reduced.  Ref: 12177064 J Biol Chem, 2002
- The RON  [ **MST1R** ]  receptor tyrosine kinase is activated by macrophage stimulating protein  [ **MST1** ] , which regulates macrophage migration, phagocytosis, and nitric oxide production.  Ref: 12177064 J Biol Chem, 2002
- The interaction of Ron  [ **MST1R** ]  with its ligand, known as hepatocyte growth factor like protein  [ **MST1** ]  HGFL  [ **MST1** ]  or macrophage stimulating protein  [ **MST1** ]  MSP  [ **MST1** ] , induces crucial cellular responses including invasive growth, proliferation, cell scattering, and branching morphogenesis.  Ref: 11593422 Oncogene, 2001
- Recently, we established IL 3 dependent Ba F3 pro B cells ectopically expressing RON  [ **MST1R** ]  tyrosine kinase, a receptor for macrophage stimulating protein  [ **MST1** ]  MSP  [ **MST1** ] , and showed that MSP  [ **MST1** ]  stimulation specifically promoted cell morphological changes through tyrosine phosphorylation of the IL 3 common beta chain receptor subunit betac by activated RON  [ **MST1R** ]  kinase without activation of JAK2 tyrosine kinase.  Ref: 12270548 Immunol Lett, 2002
**Neutral relationship**- The specific receptor for MSP  [ **MST1** ]  is the RON  [ **MST1R** ]  recepteur d origine nantais receptor tyrosine kinase a member of the MET proto oncogene family.  Ref: 12472665 Scand J Immunol, 2002
- Activation of CR3 mediated phagocytosis by MSP  [ **MST1** ]  requires the RON  [ **MST1R** ]  receptor, tyrosine kinase activity, phosphatidylinositol 3 kinase, and protein kinase C zeta.  Ref: 12773513 J Leukoc Biol, 2003
- MSP  [ **MST1** ]  is the ligand for Ron  [ **MST1R** ] , a member of the Met subfamily of tyrosine kinase receptors.  Ref: 11704649 Br J Pharmacol, 2001
- These results indicate a novel field of operation for MSP  [ **MST1** ]  and suggest a pathogenic role of the MSP  [ **MST1** ]  RON  [ **MST1R** ]  system in renal disease.  Ref: 11856768 J Am Soc Nephrol, 2002
- Consistently, the mature Ron  [ **MST1R** ]  protein is expressed by the MSP  [ **MST1** ]  responsive cells but not by the unresponsive monocytes.  Ref: 11704649 Br J Pharmacol, 2001
- MSP  [ **MST1** ]  in HK2 supernatant and rMSP induced in HMC phosphorylation of RON  [ **MST1R** ] , growth, migration, invasion, and IL 6 synthesis.  Ref: 11856768 J Am Soc Nephrol, 2002
- Ron  [ **MST1R** ]  is a receptor tyrosine kinase that is activated by the binding of hepatocyte growth factor like  [ **MST1** ]  HGFL  [ **MST1** ]  protein.  Ref: 12606483 Biol Reprod, 2003
- We show that MSP  [ **MST1** ]  induced complement mediated phagocytosis requires the RON  [ **MST1R** ]  receptor tyrosine kinase and the alphaMbeta2 integrin, as evidenced by the inability of RON  [ **MST1R** ]  and alphaM peritoneal macrophages to augment phagocytosis of complement coated sheep erythrocytes in response to MSP  [ **MST1** ] .  Ref: 12773513 J Leukoc Biol, 2003
- To begin to examine the involvement of HGF and MSP  [ **MST1** ]  in healing of cutaneous wounds we have mapped the temporal expression of these two molecules and their receptors, MET and RON  [ **MST1R** ]  respectively, in adult rat excisional wounds.  Ref: 11702235 Cell Tissue Res, 2001

**Non-interactive relationship** (e.g. studied together, co-existance, homology, etc.)

- These observations suggest that activation of the Ron  [ **MST1R** ]  receptor by HGFL  [ **MST1** ]  binding may aid in implantation by way of trophoblast function and viability.  Ref: 12606483 Biol Reprod, 2003
- To investigate whether MSP  [ **MST1** ]  is also capable of acting as a neurotrophic factor, we purified hypoglossal motoneurons from the embryonic chicken hindbrain because these neurons are known to express the MSP  [ **MST1** ]  receptor tyrosine kinase RON  [ **MST1R** ] .  Ref: 11860510 Eur J Neurosci, 2002
- Thus, MSP  [ **MST1** ]  and RON  [ **MST1R** ]  are potential regulators that control macrophage activities during bacterial infection in vivo.  Ref: 12472665 Scand J Immunol, 2002
- In normal human kidney, tubules expressed MSP  [ **MST1** ]  and RON  [ **MST1R** ] .  Ref: 11856768 J Am Soc Nephrol, 2002
- The proto oncogene product Ron  [ **MST1R** ]  is the receptor for macrophage stimulating protein  [ **MST1** ] , a scatter factor that stimulates cell proliferation, prevents apoptosis, and induces an invasive cell phenotype.  Ref: 12766581 Am J Surg Pathol, 2003
- The expression of MSP  [ **MST1** ]  and RON  [ **MST1R** ]  in normal human renal tissue was studied by immunohistochemistry.  Ref: 11856768 J Am Soc Nephrol, 2002
- In this study, we demonstrate that stimulation of murine peritoneal macrophages with MSP  [ **MST1** ]  results in the RON  [ **MST1R** ]  dependent up regulation of arginase, an enzyme associated with alternative activation that competes with iNOS for the substrate L arginine, the products of which are involved in cell proliferation and matrix synthesis.  Ref: 11777982 J Immunol, 2002
- Thus, increased expression of HGF, MSP  [ **MST1** ]  and their receptors MET and RON  [ **MST1R** ]  respectively was observed in response to wounding.  Ref: 11702235 Cell Tissue Res, 2001
- Our lab and others have previously shown that macrophage stimulating protein  [ **MST1** ]  MSP  [ **MST1** ] , through activation of its receptor RON  [ **MST1R** ] , negatively regulates NO production in response to IFN gamma and LPS by inhibiting the expression of inducible NO synthase iNOS.  Ref: 11777982 J Immunol, 2002
- The specific cellular localization and mechanisms of action of Ron  [ **MST1R** ]  and HGFL  [ **MST1** ]  during embryo implantation are not known.  Ref: 12606483 Biol Reprod, 2003
- Although  **MST1**  deficient mice are viable,  **MST1R**  is essential in mice before gastrulation for implantation, and is a known oncogene in man.  Ref: 12764615 Dev Genes Evol< MedlineTA>Dev Genes Evol, 2003
- Ron  [ **MST1R** ] , the receptor tyrosine kinase RTK for the macrophage stimulating protein  [ **MST1** ]  MSP  [ **MST1** ] , activates multiple signaling pathways by recruiting several positive regulators to a multifunctional docking site.  Ref: 12802274 Oncogene, 2003
- Macrophage stimulating protein  [ **MST1** ]  and RON  [ **MST1R** ]  receptor tyrosine kinase potential regulators of macrophage inflammatory activities.  Ref: 12472665 Scand J Immunol, 2002
- Oncogenic and invasive potentials of human macrophage stimulating protein  [ **MST1** ]  receptor, the RON  [ **MST1R** ]  receptor tyrosine kinase.  Ref: 12807733 Carcinogenesis< MedlineTA>Carcinogenesis, 2002
- Treatment of RON  [ **MST1R** ]  expressing cells with PD98059 or U0126, inhibitors of mitogen activated protein kinase kinase activity, blocked both IL 3 and MSP  [ **MST1** ]  induced morphological changes.  Ref: 12270548 Immunol Lett, 2002
- These results clearly indicate that hrMSP induces the respiratory burst in human macrophages but not in monocytes, suggesting for the MSP  [ **MST1** ]  Ron  [ **MST1R** ]  complex a role of activator as well as of possible marker for human mature macrophages.  Ref: 11704649 Br J Pharmacol, 2001
- Immunofluorescent staining revealed both MSP  [ **MST1** ]  and RON  [ **MST1R** ]  within the wound, with maximum staining occurring between 7 and 21 days for both the ligand and receptor.  Ref: 11702235 Cell Tissue Res, 2001
- Activation of RON  [ **MST1R** ]  by MSP  [ **MST1** ]  exerts dual functions on macrophages.  Ref: 12472665 Scand J Immunol, 2002
- HMC expressed RON  [ **MST1R** ]  but did not produce MSP  [ **MST1** ] .  Ref: 11856768 J Am Soc Nephrol, 2002
- The overexpression of bcl 2, but not p53 in oncocytoma, suggests that the MSP  [ **MST1** ]  Ron  [ **MST1R** ]  system sustains the growth of oncocytoma by opposing apoptosis.  Ref: 12766581 Am J Surg Pathol, 2003
- Activation of the RON  [ **MST1R** ]  receptor tyrosine kinase by macrophage stimulating protein  [ **MST1** ]  inhibits inducible cyclooxygenase 2 expression in murine macrophages.  Ref: 12177064 J Biol Chem, 2002
- HK2 produced MSP  [ **MST1** ]  and expressed RON  [ **MST1R** ]  in a form that was phosphorylated by rMSP.  Ref: 11856768 J Am Soc Nephrol, 2002
- The expression of the MSP  [ **MST1** ]  receptor, RON  [ **MST1R** ] , was evaluated in HMC and HK2 by Western blot.  Ref: 11856768 J Am Soc Nephrol, 2002
- Activation of the stem cell derived tyrosine kinase  [ **MST1R** ]  RON  [ **MST1R** ]  receptor tyrosine kinase by macrophage stimulating protein  [ **MST1** ]  results in the induction of arginase activity in murine peritoneal macrophages.  Ref: 11777982 J Immunol, 2002
- Here we present a chimeric cytokine derived from the alpha chains of HGF and MSP  [ **MST1** ] , named Metron factor 1 for its ability to concomitantly activate the HGF receptor Met and the MSP  [ **MST1** ]  receptor Ron  [ **MST1R** ] .  Ref: 11981563 Nat Biotechnol, 2002
